# Supplementary figures and images for: Male Age and Wolbachia Dynamics: Investigating How Fast and Why Bacterial Densities and Cytoplasmic Incompatibility Strengths Vary
Source: mBio. 2021 Dec 14;12(6):e02998-21. doi: 10.1128/mBio.02998-21 (PMC8686834; doi:10.1128/mBio.02998-21)

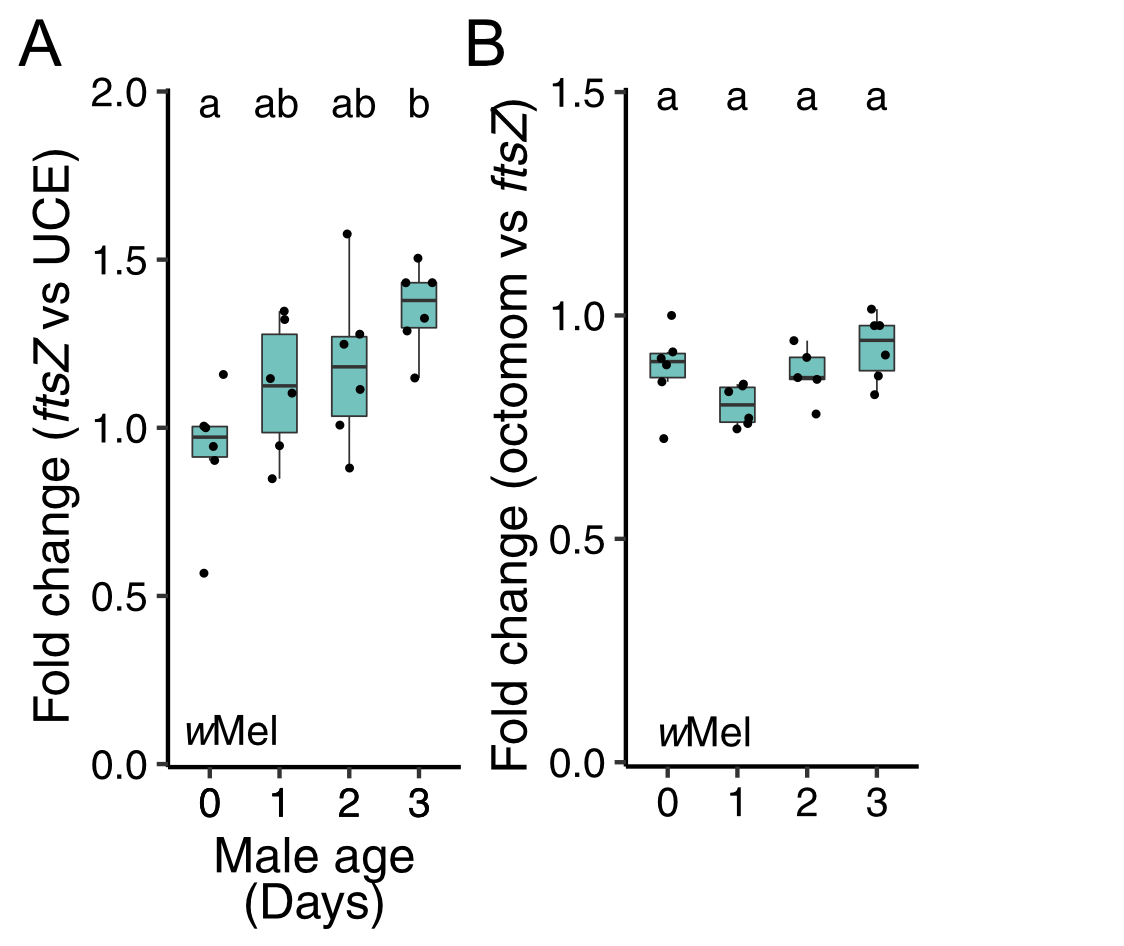

Supplement: FIG S1 [file mbio.02998-21-sf001.tif]

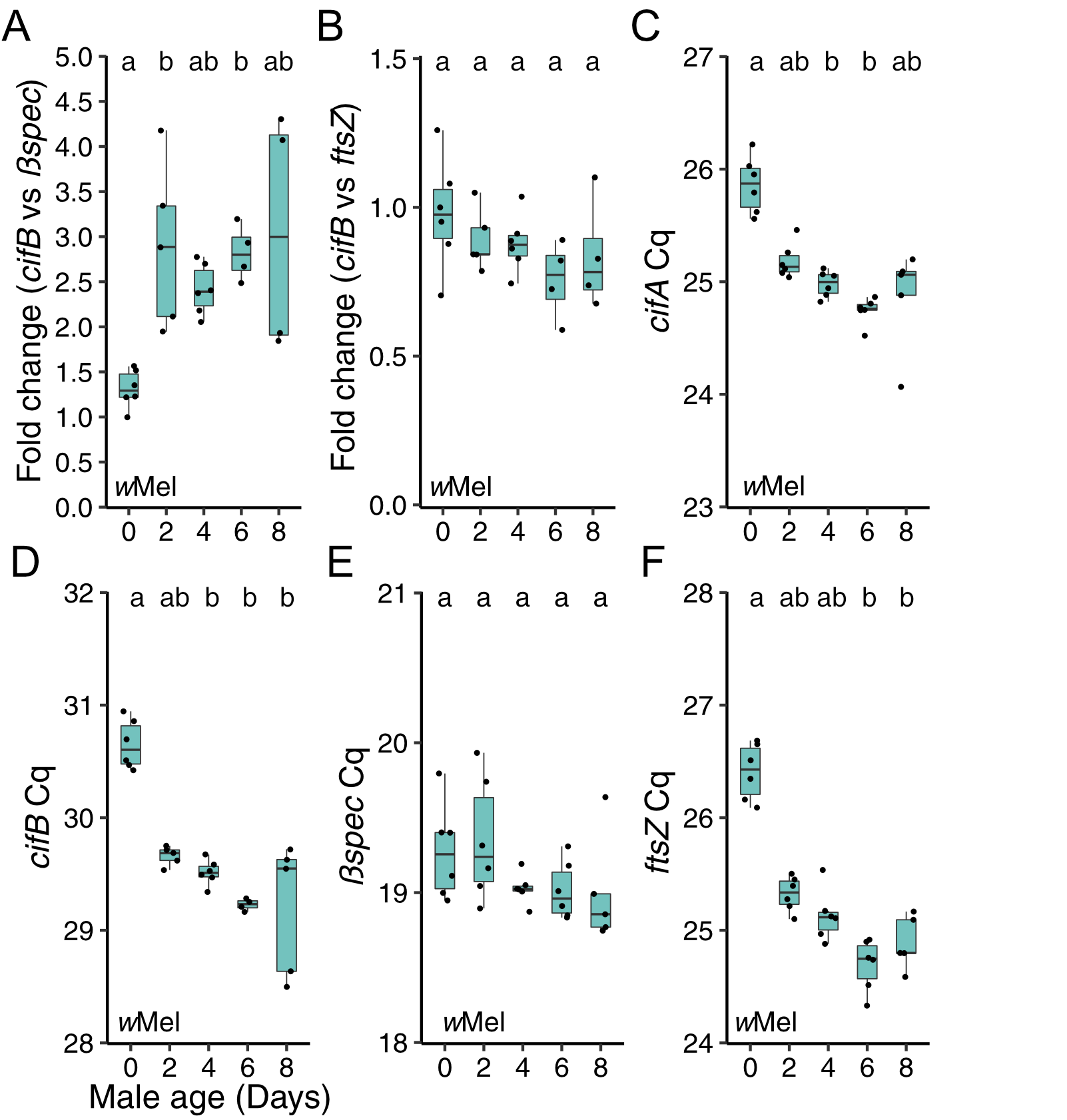

Supplement: FIG S2 [file mbio.02998-21-sf002.tif]

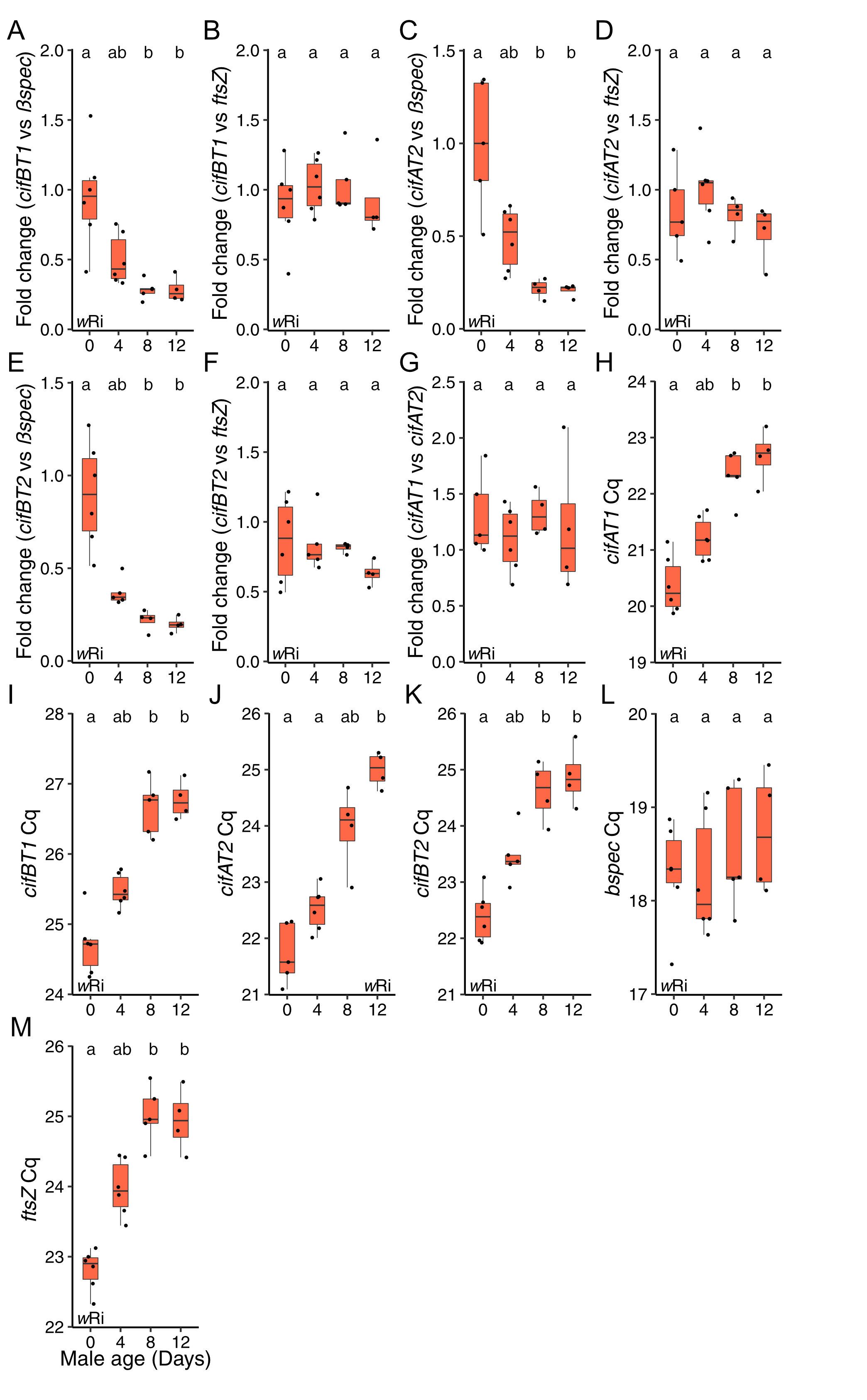

Supplement: FIG S3 [file mbio.02998-21-sf003.tif]

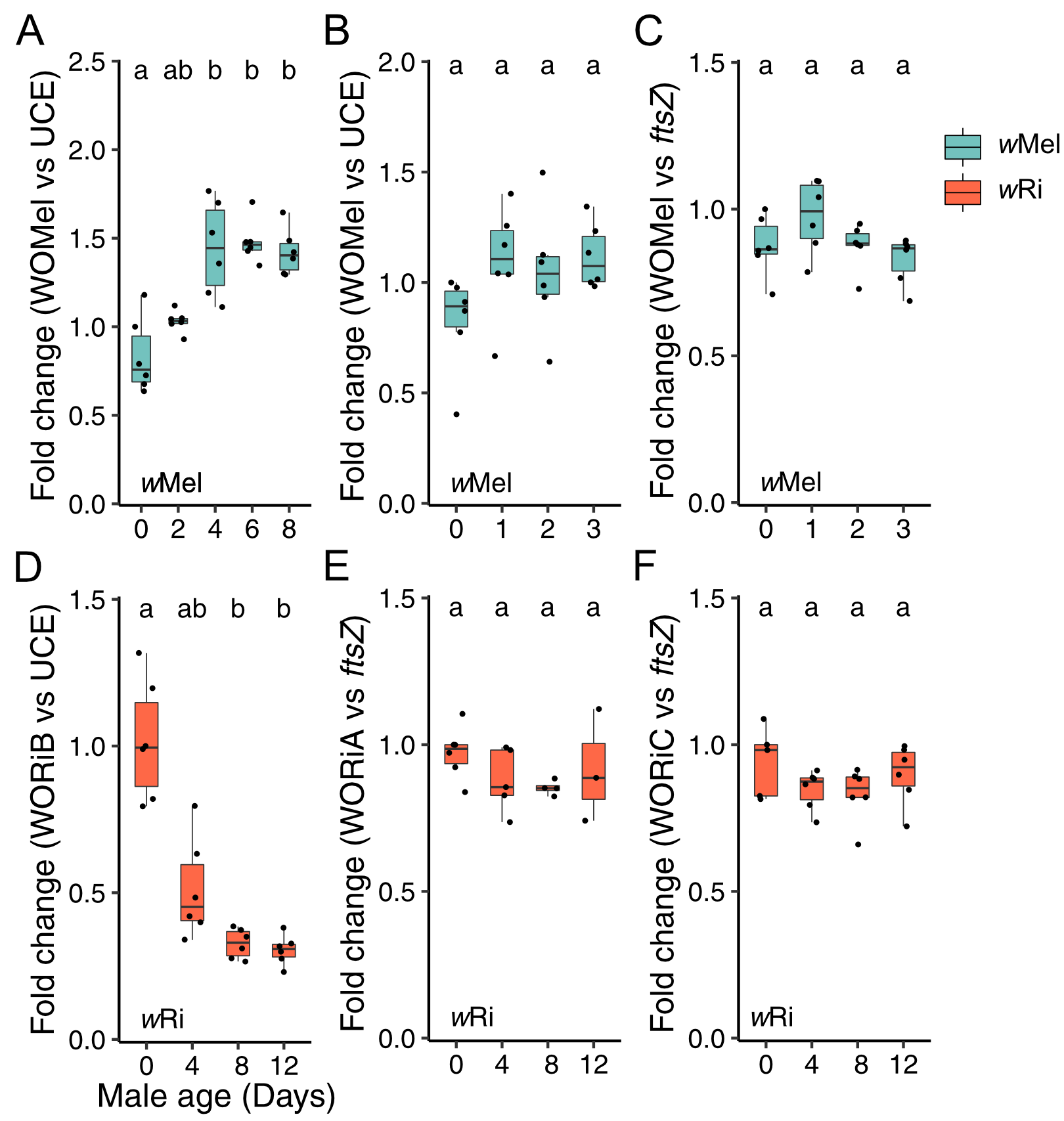

Supplement: FIG S4 [file mbio.02998-21-sf004.tif]
